# Tetra-μ-acetato-κ^8^ *O*:*O*′-bis­{[*N*-(4-methyl­phen­yl)pyridin-2-amine-κ*N* ^1^]copper(II)}(*Cu*—*Cu*)

**DOI:** 10.1107/S1600536811044333

**Published:** 2011-10-29

**Authors:** Zainal Abidin Fairuz, Zaharah Aiyub, Zanariah Abdullah, Seik Weng Ng, Edward R. T. Tiekink

**Affiliations:** a Department of Chemistry, University of Malaya, 50603 Kuala Lumpur, Malaysia; b Chemistry Department, Faculty of Science, King Abdulaziz University, PO Box 80203 Jeddah, Saudi Arabia

## Abstract

The complete dinuclear mol­ecule of the title complex, [Cu_2_(CH_3_COO)_4_(C_12_H_12_N_2_)_2_], is generated by a centre of inversion. The Cu^II^ atoms are connected [Cu—Cu = 2.6329 (16) Å] and bridged by four acetate ligands. The distorted octa­hedral coordination geometry is completed by a terminal pyridine N atom. The amine H atom forms an intra­molecular N—H⋯O hydrogen bond.

## Related literature

For related examples of tetra­kis­acetato­bis­[(substituted 2-amino­pyrid­yl)copper] complexes, see: Fairuz *et al.* (2010*a* ▶,*b* ▶).
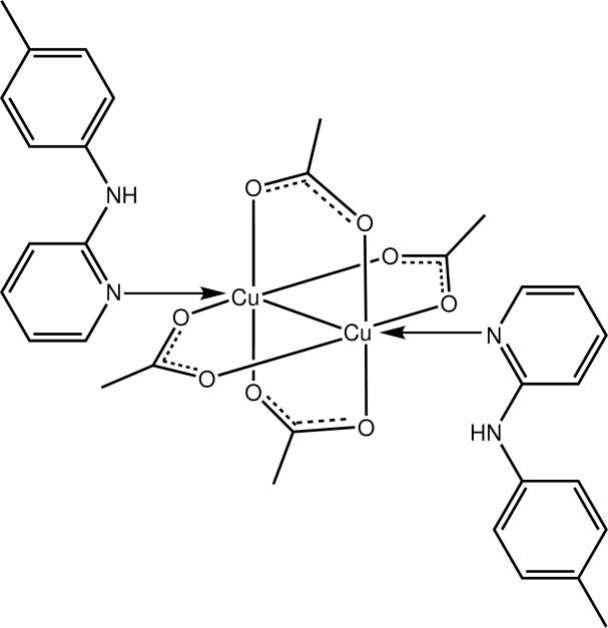


## Experimental

### 

#### Crystal data

[Cu_2_(C_2_H_3_O_2_)_4_(C_12_H_12_N_2_)_2_]
*M* *_r_* = 731.75
Monoclinic, 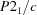

*a* = 7.6285 (9) Å
*b* = 11.3242 (13) Å
*c* = 18.566 (2) Å
β = 95.717 (2)°
*V* = 1595.9 (3) Å^3^
*Z* = 2
Mo *K*α radiation
μ = 1.39 mm^−1^
*T* = 100 K
0.22 × 0.13 × 0.05 mm

#### Data collection

Bruker SMART APEX diffractometer
Absorption correction: multi-scan (*SADABS*; Sheldrick, 1996 ▶) *T*_min_ = 0.495, *T*_max_ = 0.862
11607 measured reflections
2806 independent reflections
2203 reflections with *I* > 2σ(*I*)
*R*_int_ = 0.103

#### Refinement

*R*[*F* ^2^ > 2σ(*F* ^2^)] = 0.077
*wR*(*F* ^2^) = 0.209
*S* = 1.08
2806 reflections
211 parameters
H-atom parameters constrained
Δρ_max_ = 1.31 e Å^−3^
Δρ_min_ = −1.29 e Å^−3^

### 

Data collection: *APEX2* (Bruker, 2009 ▶); cell refinement: *SAINT* (Bruker, 2009 ▶); data reduction: *SAINT*; program(s) used to solve structure: *SHELXS97* (Sheldrick, 2008 ▶); program(s) used to refine structure: *SHELXL97* (Sheldrick, 2008 ▶); molecular graphics: *ORTEP-3* (Farrugia, 1997 ▶) and *DIAMOND* (Brandenburg, 2006 ▶); software used to prepare material for publication: *publCIF* (Westrip, 2010 ▶).

## Supplementary Material

Crystal structure: contains datablock(s) global, I. DOI: 10.1107/S1600536811044333/hg5122sup1.cif

Structure factors: contains datablock(s) I. DOI: 10.1107/S1600536811044333/hg5122Isup2.hkl

Additional supplementary materials:  crystallographic information; 3D view; checkCIF report

## Figures and Tables

**Table 1:** Selected bond lengths (Å)

Cu—O2^i^	1.947 (5)
Cu—O1	1.950 (5)
Cu—O3	1.976 (5)
Cu—O4^i^	1.976 (5)
Cu—N1	2.205 (6)

Symmetry code: (i) 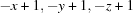.

**Table 2:** Hydrogen-bond geometry (Å, °)

*D*—H⋯*A*	*D*—H	H⋯*A*	*D*⋯*A*	*D*—H⋯*A*
N2—H2*n*⋯O3	0.86	2.21	2.911 (8)	139

**Fig. 1.**
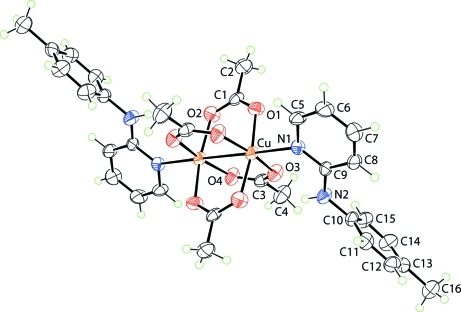
The molecular structure of (I) showing the atom-labelling scheme and displacement ellipsoids at the 50% probability level. The complex is centrosymmetric. The unlabelled atoms are related by the symmetry operation 1 - x, 1 - y, 1 - z.

## supplementary crystallographic information

### Comment

The crystal structure of the title complex, (I), was investigated in connection with structural studies of tetrakisacetatobis[(substituted 2-aminopyridyl)copper(II)] complexes (Fairuz *et al.*, 2010*a*; Fairuz *et al.*, 2010*b*). The complex, Fig. 1, is centrosymmetric and feature four symmetrically bridging acetate ligands and two terminally connected pyridyl-N atoms. These define an NO_4_ donor set and the distorted octahedral geometry is completed by a Cu atom, Table 1. The orientation of the *N-p*-tolylpyridin-2-amine ligand is such to enable the formation of an intramolecular N—H···O hydrogen bond, Table 2. The pyridyl-2-amine ligand is twisted with the dihedral angle between the pyridyl and benzene rings being 59.3 (4)°.

### Experimental

*N-p*-Tolylpyridin-2-amine (0.2 g, 1.1 mmol) was dissolved in acetonitrile (15 ml), added to trimethyl orthoformate (10 ml) and the mixture then heated to 50 °C. Copper acetate (0.1 g, 0.5 mmol) dissolved in acetonitrile (15 ml) was added to the solution. The green precipitate that formed, was collected and recrystallized from acetonitrile to give green crystals.

### Refinement

Hydrogen atoms were placed at calculated positions (C—H 0.95–098 Å, N–H 0.86 Å) and were treated as riding on their parent carbon atoms, with *U*(H) set to 1.2–1.5 times *U*_eq_(C). The maximum and minimum residual electron density peaks of 1.31 and 1.89 e Å^-3^, respectively, were located 1.08 Å and 0.91 Å from the Cu atom.

### Crystal data

**Table:**

[Cu_2_(C_2_H_3_O_2_)_4_(C_12_H_12_N_2_)_2_]	*F*(000) = 756
*M**_r_* = 731.75	*D*_x_ = 1.523 Mg m^−^^3^
Monoclinic, *P*2_1_/*c*	Mo *K*α radiation, λ = 0.71073 Å
Hall symbol: -P 2ybc	Cell parameters from 2708 reflections
*a* = 7.6285 (9) Å	θ = 2.7–23.1°
*b* = 11.3242 (13) Å	µ = 1.39 mm^−^^1^
*c* = 18.566 (2) Å	*T* = 100 K
β = 95.717 (2)°	Prism, green
*V* = 1595.9 (3) Å^3^	0.22 × 0.13 × 0.05 mm
*Z* = 2	

### Data collection

**Table:**

Bruker SMART APEX diffractometer	2806 independent reflections
Radiation source: fine-focus sealed tube	2203 reflections with *I* > 2σ(*I*)
graphite	*R*_int_ = 0.103
ω scans	θ_max_ = 25.0°, θ_min_ = 2.1°
Absorption correction: multi-scan (*SADABS*; Sheldrick, 1996)	*h* = −9→9
*T*_min_ = 0.495, *T*_max_ = 0.862	*k* = −13→13
11607 measured reflections	*l* = −22→22

### Refinement

**Table:**

Refinement on *F*^2^	Primary atom site location: structure-invariant direct methods
Least-squares matrix: full	Secondary atom site location: difference Fourier map
*R*[*F*^2^ > 2σ(*F*^2^)] = 0.077	Hydrogen site location: inferred from neighbouring sites
*wR*(*F*^2^) = 0.209	H-atom parameters constrained
*S* = 1.08	*w* = 1/[σ^2^(*F*_o_^2^) + (0.0765*P*)^2^ + 11.519*P*] where *P* = (*F*_o_^2^ + 2*F*_c_^2^)/3
2806 reflections	(Δ/σ)_max_ = 0.004
211 parameters	Δρ_max_ = 1.31 e Å^−^^3^
0 restraints	Δρ_min_ = −1.29 e Å^−^^3^

### Fractional atomic coordinates and isotropic or equivalent isotropic displacement parameters (Å^2^)

**Table:**

	*x*	*y*	*z*	*U*_iso_*/*U*_eq_	
Cu	0.59639 (11)	0.59398 (7)	0.51769 (5)	0.0291 (3)	
O1	0.7488 (7)	0.4818 (4)	0.5735 (3)	0.0422 (14)	
O2	0.5861 (7)	0.3220 (5)	0.5427 (3)	0.0424 (14)	
O3	0.4444 (7)	0.6035 (5)	0.5977 (3)	0.0408 (13)	
O4	0.2853 (7)	0.4450 (5)	0.5692 (3)	0.0426 (14)	
N1	0.7623 (8)	0.7488 (5)	0.5486 (3)	0.0301 (13)	
N2	0.5402 (8)	0.8521 (6)	0.5941 (4)	0.0422 (17)	
H2n	0.4726	0.7948	0.5785	0.051*	
C1	0.7159 (9)	0.3738 (6)	0.5757 (4)	0.0323 (17)	
C2	0.8381 (13)	0.2988 (8)	0.6240 (5)	0.052 (2)	
H2A	0.8227	0.2158	0.6099	0.079*	
H2B	0.8119	0.3085	0.6743	0.079*	
H2C	0.9601	0.3227	0.6196	0.079*	
C3	0.3262 (10)	0.5337 (6)	0.6077 (4)	0.0330 (17)	
C4	0.2223 (13)	0.5545 (8)	0.6707 (5)	0.052 (2)	
H4A	0.1985	0.6391	0.6750	0.078*	
H4B	0.2897	0.5265	0.7151	0.078*	
H4C	0.1105	0.5113	0.6633	0.078*	
C5	0.9295 (9)	0.7373 (7)	0.5348 (4)	0.0377 (18)	
H5	0.9643	0.6655	0.5139	0.045*	
C6	1.0509 (10)	0.8218 (8)	0.5488 (5)	0.047 (2)	
H6	1.1689	0.8111	0.5378	0.056*	
C7	0.9980 (11)	0.9249 (7)	0.5798 (5)	0.047 (2)	
H7	1.0804	0.9869	0.5903	0.057*	
C8	0.8305 (10)	0.9382 (7)	0.5952 (5)	0.0388 (19)	
H8	0.7948	1.0090	0.6170	0.047*	
C9	0.7114 (9)	0.8485 (6)	0.5790 (4)	0.0292 (16)	
C10	0.4654 (10)	0.9431 (7)	0.6335 (4)	0.0345 (17)	
C11	0.4545 (10)	1.0572 (7)	0.6082 (4)	0.0374 (18)	
H11	0.5019	1.0777	0.5644	0.045*	
C12	0.3738 (10)	1.1416 (7)	0.6472 (4)	0.0395 (18)	
H12	0.3673	1.2204	0.6296	0.047*	
C13	0.3027 (10)	1.1160 (7)	0.7101 (4)	0.0376 (18)	
C14	0.3148 (11)	0.9995 (8)	0.7341 (4)	0.045 (2)	
H14	0.2649	0.9779	0.7771	0.054*	
C15	0.3975 (11)	0.9159 (7)	0.6965 (5)	0.044 (2)	
H15	0.4076	0.8375	0.7147	0.053*	
C16	0.2120 (13)	1.2061 (9)	0.7533 (5)	0.058 (2)	
H16A	0.2487	1.1952	0.8050	0.087*	
H16B	0.0841	1.1961	0.7444	0.087*	
H16C	0.2443	1.2857	0.7387	0.087*	

### Atomic displacement parameters (Å^2^)

**Table:**

	*U*^11^	*U*^22^	*U*^33^	*U*^12^	*U*^13^	*U*^23^
Cu	0.0282 (5)	0.0182 (5)	0.0409 (5)	−0.0059 (4)	0.0038 (3)	−0.0019 (4)
O1	0.034 (3)	0.023 (3)	0.068 (4)	0.000 (2)	−0.007 (3)	0.000 (2)
O2	0.042 (3)	0.023 (3)	0.060 (4)	−0.009 (2)	−0.004 (3)	0.006 (2)
O3	0.044 (3)	0.029 (3)	0.052 (3)	−0.009 (3)	0.017 (3)	−0.002 (2)
O4	0.039 (3)	0.033 (3)	0.058 (4)	−0.014 (3)	0.016 (3)	−0.011 (3)
N1	0.028 (3)	0.021 (3)	0.041 (3)	−0.009 (2)	0.002 (3)	0.001 (3)
N2	0.028 (3)	0.026 (3)	0.073 (5)	−0.008 (3)	0.007 (3)	−0.015 (3)
C1	0.027 (4)	0.019 (4)	0.052 (5)	−0.001 (3)	0.013 (3)	−0.003 (3)
C2	0.057 (6)	0.031 (5)	0.067 (6)	0.008 (4)	−0.005 (5)	0.000 (4)
C3	0.035 (4)	0.022 (4)	0.043 (4)	0.002 (3)	0.007 (3)	0.005 (3)
C4	0.058 (6)	0.040 (5)	0.062 (6)	−0.004 (4)	0.023 (5)	−0.005 (4)
C5	0.020 (4)	0.034 (4)	0.060 (5)	0.000 (3)	0.008 (3)	−0.005 (4)
C6	0.020 (4)	0.044 (5)	0.076 (6)	−0.008 (4)	0.004 (4)	−0.006 (4)
C7	0.033 (4)	0.035 (5)	0.073 (6)	−0.018 (4)	0.004 (4)	−0.007 (4)
C8	0.033 (4)	0.023 (4)	0.060 (5)	−0.003 (3)	0.000 (4)	−0.009 (3)
C9	0.034 (4)	0.017 (3)	0.036 (4)	−0.002 (3)	−0.002 (3)	−0.002 (3)
C10	0.031 (4)	0.025 (4)	0.047 (5)	−0.002 (3)	0.001 (3)	−0.008 (3)
C11	0.036 (4)	0.030 (4)	0.046 (5)	−0.011 (3)	0.008 (3)	0.000 (3)
C12	0.040 (4)	0.027 (4)	0.052 (5)	−0.002 (3)	0.003 (4)	0.004 (4)
C13	0.038 (4)	0.030 (4)	0.044 (4)	0.000 (3)	−0.006 (3)	−0.006 (3)
C14	0.054 (5)	0.040 (5)	0.044 (5)	0.007 (4)	0.016 (4)	0.009 (4)
C15	0.053 (5)	0.023 (4)	0.058 (5)	0.002 (4)	0.007 (4)	0.010 (4)
C16	0.060 (6)	0.048 (6)	0.067 (6)	0.009 (5)	0.005 (5)	−0.016 (5)

### Geometric parameters (Å, °)

**Table:**

Cu—O2^i^	1.947 (5)	C4—H4C	0.9800
Cu—O1	1.950 (5)	C5—C6	1.339 (11)
Cu—O3	1.976 (5)	C5—H5	0.9500
Cu—O4^i^	1.976 (5)	C6—C7	1.380 (12)
Cu—N1	2.205 (6)	C6—H6	0.9500
Cu—Cu^i^	2.6329 (16)	C7—C8	1.345 (11)
O1—C1	1.249 (9)	C7—H7	0.9500
O2—C1	1.257 (9)	C8—C9	1.376 (10)
O2—Cu^i^	1.947 (5)	C8—H8	0.9500
O3—C3	1.227 (9)	C10—C15	1.361 (11)
O4—C3	1.254 (9)	C10—C11	1.375 (11)
O4—Cu^i^	1.976 (5)	C11—C12	1.381 (11)
N1—C5	1.332 (9)	C11—H11	0.9500
N1—C9	1.337 (9)	C12—C13	1.368 (11)
N2—C9	1.363 (10)	C12—H12	0.9500
N2—C10	1.417 (10)	C13—C14	1.393 (11)
N2—H2n	0.8600	C13—C16	1.508 (11)
C1—C2	1.494 (11)	C14—C15	1.368 (12)
C2—H2A	0.9800	C14—H14	0.9500
C2—H2B	0.9800	C15—H15	0.9500
C2—H2C	0.9800	C16—H16A	0.9800
C3—C4	1.496 (11)	C16—H16B	0.9800
C4—H4A	0.9800	C16—H16C	0.9800
C4—H4B	0.9800		
			
O2^i^—Cu—O1	168.3 (2)	H4A—C4—H4C	109.5
O2^i^—Cu—O3	88.1 (2)	H4B—C4—H4C	109.5
O1—Cu—O3	90.0 (2)	N1—C5—C6	123.4 (8)
O2^i^—Cu—O4^i^	89.8 (3)	N1—C5—H5	118.3
O1—Cu—O4^i^	89.5 (3)	C6—C5—H5	118.3
O3—Cu—O4^i^	167.4 (2)	C5—C6—C7	117.4 (7)
O2^i^—Cu—N1	96.9 (2)	C5—C6—H6	121.3
O1—Cu—N1	94.8 (2)	C7—C6—H6	121.3
O3—Cu—N1	97.2 (2)	C8—C7—C6	120.5 (7)
O4^i^—Cu—N1	95.3 (2)	C8—C7—H7	119.8
O2^i^—Cu—Cu^i^	84.36 (16)	C6—C7—H7	119.8
O1—Cu—Cu^i^	83.94 (16)	C7—C8—C9	119.4 (7)
O3—Cu—Cu^i^	82.90 (16)	C7—C8—H8	120.3
O4^i^—Cu—Cu^i^	84.55 (16)	C9—C8—H8	120.3
N1—Cu—Cu^i^	178.73 (17)	N1—C9—N2	115.8 (6)
C1—O1—Cu	122.9 (5)	N1—C9—C8	120.2 (7)
C1—O2—Cu^i^	122.4 (5)	N2—C9—C8	124.0 (7)
C3—O3—Cu	125.0 (5)	C15—C10—C11	119.5 (7)
C3—O4—Cu^i^	122.4 (5)	C15—C10—N2	119.1 (7)
C5—N1—C9	119.2 (6)	C11—C10—N2	121.3 (7)
C5—N1—Cu	114.1 (5)	C10—C11—C12	119.0 (7)
C9—N1—Cu	126.7 (5)	C10—C11—H11	120.5
C9—N2—C10	124.8 (6)	C12—C11—H11	120.5
C9—N2—H2n	117.6	C13—C12—C11	122.5 (7)
C10—N2—H2n	117.6	C13—C12—H12	118.7
O1—C1—O2	126.3 (7)	C11—C12—H12	118.7
O1—C1—C2	117.4 (7)	C12—C13—C14	117.0 (7)
O2—C1—C2	116.2 (7)	C12—C13—C16	123.6 (8)
C1—C2—H2A	109.5	C14—C13—C16	119.4 (8)
C1—C2—H2B	109.5	C15—C14—C13	120.8 (8)
H2A—C2—H2B	109.5	C15—C14—H14	119.6
C1—C2—H2C	109.5	C13—C14—H14	119.6
H2A—C2—H2C	109.5	C10—C15—C14	121.1 (7)
H2B—C2—H2C	109.5	C10—C15—H15	119.5
O3—C3—O4	125.1 (7)	C14—C15—H15	119.5
O3—C3—C4	118.2 (7)	C13—C16—H16A	109.5
O4—C3—C4	116.7 (7)	C13—C16—H16B	109.5
C3—C4—H4A	109.5	H16A—C16—H16B	109.5
C3—C4—H4B	109.5	C13—C16—H16C	109.5
H4A—C4—H4B	109.5	H16A—C16—H16C	109.5
C3—C4—H4C	109.5	H16B—C16—H16C	109.5
			
O2^i^—Cu—O1—C1	2.3 (16)	C9—N1—C5—C6	−1.1 (12)
O3—Cu—O1—C1	82.8 (6)	Cu—N1—C5—C6	179.3 (7)
O4^i^—Cu—O1—C1	−84.7 (6)	N1—C5—C6—C7	0.7 (14)
N1—Cu—O1—C1	−180.0 (6)	C5—C6—C7—C8	0.2 (14)
Cu^i^—Cu—O1—C1	−0.1 (6)	C6—C7—C8—C9	−0.7 (14)
O2^i^—Cu—O3—C3	84.9 (6)	C5—N1—C9—N2	−177.6 (7)
O1—Cu—O3—C3	−83.6 (6)	Cu—N1—C9—N2	2.1 (9)
O4^i^—Cu—O3—C3	4.2 (15)	C5—N1—C9—C8	0.5 (11)
N1—Cu—O3—C3	−178.4 (6)	Cu—N1—C9—C8	−179.8 (5)
Cu^i^—Cu—O3—C3	0.3 (6)	C10—N2—C9—N1	172.4 (7)
O2^i^—Cu—N1—C5	−125.9 (5)	C10—N2—C9—C8	−5.6 (13)
O1—Cu—N1—C5	54.6 (6)	C7—C8—C9—N1	0.3 (12)
O3—Cu—N1—C5	145.2 (5)	C7—C8—C9—N2	178.3 (8)
O4^i^—Cu—N1—C5	−35.4 (6)	C9—N2—C10—C15	−118.9 (9)
O2^i^—Cu—N1—C9	54.5 (6)	C9—N2—C10—C11	63.5 (11)
O1—Cu—N1—C9	−125.0 (6)	C15—C10—C11—C12	−0.3 (12)
O3—Cu—N1—C9	−34.5 (6)	N2—C10—C11—C12	177.3 (7)
O4^i^—Cu—N1—C9	145.0 (6)	C10—C11—C12—C13	−0.5 (12)
Cu—O1—C1—O2	1.2 (11)	C11—C12—C13—C14	0.0 (12)
Cu—O1—C1—C2	−176.5 (6)	C11—C12—C13—C16	−179.3 (8)
Cu^i^—O2—C1—O1	−1.9 (11)	C12—C13—C14—C15	1.2 (12)
Cu^i^—O2—C1—C2	175.9 (6)	C16—C13—C14—C15	−179.4 (8)
Cu—O3—C3—O4	1.0 (12)	C11—C10—C15—C14	1.6 (13)
Cu—O3—C3—C4	−179.5 (6)	N2—C10—C15—C14	−176.1 (8)
Cu^i^—O4—C3—O3	−2.1 (11)	C13—C14—C15—C10	−2.0 (14)
Cu^i^—O4—C3—C4	178.3 (6)		

Symmetry codes: (i) −*x*+1, −*y*+1, −*z*+1.

### Hydrogen-bond geometry (Å, °)

**Table:**

*D*—H···*A*	*D*—H	H···*A*	*D*···*A*	*D*—H···*A*
N2—H2n···O3	0.86	2.21	2.911 (8)	139
